# Molecular Research in Pancreatic Cancer: Small Molecule Inhibitors, Their Mechanistic Pathways and Beyond

**DOI:** 10.3390/cimb45030124

**Published:** 2023-02-27

**Authors:** Shaila A. Shetu, Nneoma James, Gildardo Rivera, Debasish Bandyopadhyay

**Affiliations:** 1 Department of Chemistry, The University of Texas Rio Grande Valley, 1201 West University Drive, Edinburg, TX 78539, USA; 2 Laboratorio de Biotecnología Farmacéutica, Centro de Biotecnología Genómica, Instituto Politécnico Nacional, Reynosa 88710, Mexico; 3 School of Earth Environment & Marine Sciences (SEEMS), The University of Texas Rio Grande Valley, 1201 West University Drive, Edinburg, TX 78539, USA

**Keywords:** Kirsten rat sarcoma oncogene homolog (KRAS), CDKN2A, TP53, SMAD4, pancreatic cancer, pancreatic ductal adenocarcinoma, small molecule inhibitors, natural inhibitors, thymidylate synthase, pancreatic cancer drugs

## Abstract

Pancreatic enzymes assist metabolic digestion, and hormones like insulin and glucagon play a critical role in maintaining our blood sugar levels. A malignant pancreas is incapable of doing its regular functions, which results in a health catastrophe. To date, there is no effective biomarker to detect early-stage pancreatic cancer, which makes pancreatic cancer the cancer with the highest mortality rate of all cancer types. Primarily, mutations of the *KRAS*, *CDKN2A*, *TP53*, and *SMAD4* genes are responsible for pancreatic cancer, of which mutations of the *KRAS* gene are present in more than 80% of pancreatic cancer cases. Accordingly, there is a desperate need to develop effective inhibitors of the proteins that are responsible for the proliferation, propagation, regulation, invasion, angiogenesis, and metastasis of pancreatic cancer. This article discusses the effectiveness and mode of action at the molecular level of a wide range of small molecule inhibitors that include pharmaceutically privileged molecules, compounds under clinical trials, and commercial drugs. Both natural and synthetic small molecule inhibitors have been counted. Anti-pancreatic cancer activity and related benefits of using single and combined therapy have been discussed separately. This article sheds light on the scenario, constraints, and future aspects of various small molecule inhibitors for treating pancreatic cancer—the most dreadful cancer so far.

## 1. Introduction

Pancreatic cancer (PC) is one of the leading causes of cancer-related death all over the world. Statistics show that only around 9% of people have a chance of surviving pancreatic cancer for five years or more [1]. With the advancement of cancer research, many types of cancer are curable if diagnosed in the early stages. However, what makes pancreatic cancer so lethal is that it does not show any symptoms in the early stages, making it very difficult or impossible to be cured. It is frequently discovered at a late stage when treatment is often infeasible. Histopathologically, PC is divided into two types: exocrine, which is formed from exocrine pancreatic cells and accounts for more than 95% of all PC diagnoses, and endocrine, which is generated from hormone-producing endocrine cells [2]. PDAC (pancreatic ductal adenocarcinoma) accounts for over 90% of all exocrine tumors. PC has more recently been subclassified by genomic analysis as the squamous, pancreatic progenitor, immunogenic, and aberrantly differentiated endocrine exocrine (ADEX) [3]. The only potentially curative form of treatment for PC patients is surgical resection. Less than 20% of patients, however, have a disease state that is appropriate for this treatment due to either late diagnosis, tumor resistance to current medications, or a lack of efficient early-detection biomarkers [4]. For most cancer patients, metastases are the main factor in their death. Even with clear resection margins, over 30% of patients die from metastatic PC within 12 months of surgical resection [5,6]. Adjuvant chemotherapy and radiation have been employed, but there has been little improvement in patient survival over the last two decades [7,8]. The highly aggressive, metastatic, and complex nature of this disease poses a major barrier to improving patient outcomes.

## 2. Major Drivers of Pancreatic Cancer

Pancreatic cancer is considered the most common and most lethal type of cancer. It is the seventh most common cause of cancer-associated deaths around the world and the third leading cause of cancer-related death for both men and women in the United States. It was the only major cancer with a 5-year relative survival rate in the single digits (8.5%) [9,10], and very recently it has been increased to 11.5%. Most pancreatic malignancies arise from microscopic, non-invasive epithelial proliferation known as pancreatic intraepithelial neoplasia, which develops inside the pancreatic ducts. Among different types of pancreatic cancer, pancreatic ductal adenocarcinoma (PDAC) is the most abundant, causing 432,242 deaths globally in 2018 [1]. PDAC is a condition with genetic and epigenetic components that contribute to its onset, progression, and emergence of treatment resistance. A multistep process leads to the development and progression of pancreatic cancer. The four main driver genes commonly observed in pancreatic cancer are *KRAS* (Kirsten rat sarcoma virus), *CDKN2A* (Cyclin-dependent kinase inhibitor 2A), *TP53* (Tumor protein 53), and *SMAD4*. Analyzing the functions of these genes will help understand the core signaling pathways in pancreatic cancer [11]. Pancreatic intraepithelial neoplasia (PanIN) lesions are precursors to pancreatic cancer, which progresses to invasive carcinoma [12,13]. PanIN lesions are further subdivided into low (PanIN-1A/B) to high (PanIN-3) grade lesions based on the degree of cellular and nuclear atypia [14]. Over time, numerous genetic changes cause histologic progression through the PanIN stages (PanIN1–3), ultimately leading to invasive adenocarcinoma. These changes include microRNAs (miRNAs), various genes that promote or suppress tumor growth, and genetic mutations [15,16]. Kirsten rat sarcoma oncogene homolog (*KRAS*) is mutated, oncogenic miRNAs are overexpressed, and stromal associated factors are activated in the early stage of PanIN-1 lesions. In intermediate lesions (PanIN-2), overexpression of Mucin 1 (MUC1) and inactivation of p16/CDKN2A occur. Finally, high-grade lesions (PanIN-3) that occur at a late stage are linked to inactivating mutations in the tumor protein p53 (TP53) and breast cancer type 2 susceptibility protein (BRCA2) [13,17,18,19]. Various small molecule inhibitors are used in combination or alone for the treatment of pancreatic cancer.

## 3. KRAS Inhibitors in Pancreatic Cancer

*KRAS* gene mutations are present in more than 80% of pancreatic cancer cases at the time of diagnosis. Pancreatic ductal adenocarcinoma (PDAC) is the result of 90% of pancreatic malignancies, which develop in the exocrine compartment of the pancreas. Moreover, 98% of PDAC mutations result from codon 12 mutations. Notably, *KRAS* mutations occur mainly in codons 12, 13, or 61. The most common types of *KRAS* mutation are G12C, G12V, and G12D. Investigation found that the most aggressive PDAC subtypes of *KRAS* mutation are G12D (51%), G12V (30%), G12A/C/S (2% each), and G12L/F (1%) [20]. *KRAS* plays a vital role in the development of pancreatic cancer of the PDAC subtype [21]. Loss of the tumor suppressor gene cyclin-dependent kinase inhibitor 2A (CDKN2A) causes PDAC progression [22]. Even though the *KRAS* mutation does not have any anti-KRAS therapy and is considered undruggable, certain inhibitors have been shown to be effective against cancer. One of the most effective ways to prevent *KRAS* mutations is to block the RAF-MEK-ERK protein kinase pathway. This pathway is crucial for the development of PDAC. [23]. It is also reported that iExosomes (engineered exosomes to carry siRNA or shRNA specific to oncogenic KRAS^G12D^) inhibit PDAC in mice by delivering RNAi. However, exosomes, which are small extracellular vesicles (40–150 nm) released during the fusion of multivesicular bodies with the plasma membrane, have therapeutic potential to control KRAS-dependent pancreatic cancer [24,25]. Additionally, a slow-release biodegradable polymer matrix can be used to prevent the *KRAS* mutation. This process works by delivering siRNA as an extended-release drug to mutated *KRAS* [26]. These vaccines comprise many mutant genes that are effective against *KRAS* mutations [27,28]. The RAS inhibitors in the treatment of pancreatic cancer in shown in Table 1 [29,30,31,32,33,34,35,36,37,38].

### 3.1. AMG 510 (Lumakras or Sotorasib)

AMG 510 (Table 1) suppresses the KRAS^G12C^ oncogene by an irreversible approach involving the His95 groove, which is proximal to the cystine pocket. It also inhibits the MAPK signaling pathway in pancreatic and lung cancer. However, it had no effect on the wild-type KRAS^G12C^ mutation, demonstrating the specificity of AMG 510. Following the subsequent successes, additional research was carried out to obtain a more effective result. AMG 510 is more effective when combined with the MAPK inhibitor carboplatin than when used alone. As a result, synergistic effects on the mouse model were investigated, and 90% of animals qualified for complete tumor suppression. Finally, preliminary clinical research using a human model showed a 50% tumor reduction. However, most of the success of these substantial investigations is limited to the KRAS^G12C^ oncogene, which is predominantly prevalent in lung cancer [39]. Only 2% of PDAC is responsible for the oncogene KRAS^G12C^ [40].

### 3.2. MRTX849 (Adagrasib)

In the case of pancreatic and lung cancer, Jill Hallin et al. [41] discovered a very powerful, selective, and covalent KRAS^G12C^ inhibitor that is physically and functionally like the AMG 510. This compound has outstanding efficacy when used alone and is beneficial in combination therapy. Thus, phase I clinical research using a single drug in two patients with advanced lung and colon cancer was carried out. The results for these two serious tumors only indicated a partial response. However, the MRTX849 (Table 1) response is short-lived and ineffectual due to the resurgence of ERK signaling and the absence of mTOR-S6 signaling suppression. The results of this study raise expectations for pancreatic cancer treatment.

### 3.3. KRAS Inhibitor 11 [2-[4-(2-Methyl-3,5-Diphenylpyrazolo [1,5-a]Pyrimidin-7-yl)Piperazin-1-yl]Ethanol]

According to recent studies, AMG 510 is not the only small molecule inhibitor that is effective against the *KRAS* mutation. A novel small molecule inhibitor with the ability to bind the allosteric binding site was developed by McCarthy et al. [42]. This allosteric pyrazolopy-rimidine-based inhibitor (†††) inhibits both wild-type and *KRAS* mutant by binding to the allosteric p1 pocket. This novel small molecule inhibits the MAPK pathway by suppressing Raf signaling in response to the RAS mutation. The fact that this allosteric inhibition is not restricted to just one particular type of *KRAS* allele. It demonstrates an advantage for the wild-type and GTP-bound subtypes of the *KRAS* oncogene. Due to its remarkably great outcome, this allosteric inhibitor is now the first-line therapy for the treatment of tumors in a distinct range. This small molecule allosteric inhibitor demonstrated remarkable efficacy in lung and oral cancer cell lines, making the allosteric pyrazolopyrimidine-based inhibitor suitable for PDAC treatment.

### 3.4. Deltarasin

Recent research revealed that Deltarasin (Table 1) inhibits Phosphodiesterase-δ (PDEδ) interaction with the hydrophobic pocket of PDEδ by downregulating the RAS/RAF signaling pathway, which in turn inhibits *KRAS*- associated pancreatic ductal cancer (PDAC) [43,44]. The majority of lung cancer cases are caused by *KRAS* mutations [45]. Consequently, Leung et al. [46] were the first to discover that deltarasin effectively promotes apoptosis in lung cancer cells, both in vitro and in vivo. By blocking the MAPK/mTOR signaling pathway, it also causes autophagy in lung cancer cells. Deltarasin is also demonstrated to improve autophagic characteristics and produce more intracellular ROS when treated with 3-MA (an autophagy inhibitor), thereby protecting further autophagy.

### 3.5. Talniflumate

One of the main factors impeding the delivery of drugs is mucin, which has been found to be overexpressed in KRAS-driven pancreatic cancer in mice and human models [47,48,49,50]. A novel core mucin synthesizing enzyme, known as enzyme 2 β-1,6 N-acetylglucosaminyltransferase (GCNT3), has been identified, and its inhibition may reduce mucin overexpression [51]. Pancreatic intraepithelial neoplasia (PanIN) and PDAC exhibit higher mucin concentrations due to the *KRAS* mutation with p48Cre/+-LSL-KrasG12D/+ GEM. Further research revealed that pancreatic cancer exhibits more mucin formation due to aberrant GCNT3 enzyme expression from GEM in pancreatic cancer than the normal pancreas. In order to prevent mucin overexpression in pancreatic cancer, GCNT3 is used as a novel target. Following the in-silico validation, talniflumate was found to be a mucin inhibitor with excellent GCNT3 binding capacity. When compared to the well-known ligand GALB1, 3GALNAC, the docking score of talniflumate is particularly excellent. More research has confirmed that talniflumate suppresses GNCT3 protein expression after binding with GCNT3 and significantly reduces mucin overexpression. Additional research on the EGFR inhibitor (gefitinib) reveals that there has been a notable tumor regression in PDAC and PanIN, along with a considerable reduction in mucin expression.

### 3.6. MDC-1016

Mackenzie et al. [34] synthesized phospho-farnesylthiosalicylic acid (PFTS; MDC-1016) and tested its effectiveness, safety, and metabolism in preclinical models of pancreatic cancer. The active form of Ras, Ras-GTP, is ultimately inhibited by PFTS, which downregulates c-RAF, mitogen-activated protein-extracellular signal-regulated kinase (MEK), ERK1/2 kinase, phosphatidylinositol 3-kinase, and AKT both in vitro and in vivo. STAT3 inhibitor, which exhibits synergy in the suppression of pancreatic cancer growth. In addition, the combination of PFTS and phospho-valproic acid, a new STAT3 (signal transducer and activator of transcription 3) inhibitor, has been shown to exhibit synergistic effects in pancreatic cancer.

### 3.7. Simvastatin

Mevalonate intermediates, including farnesyl pyrophosphate (FPP) and geranylgeranyl pyrophosphate (GGPP), which are responsible for activating RAS proteins, are potentially inhibited by statins in pancreatic cancer [52]. Simvastatin (Table 1) treatment on MiaPaCa-2 human pancreatic cancer cells showed 200 genes affected by simvastatin treatment. The reason is due to the interaction with FPP and simvastatin. However, it is observed that the normalization of expression of KRAS-related gene and the GFP-K-Ras protein trafficking was partially prevented by the addition of any of the mevalonate pathway’s intermediates. Finally, the addition of FPP or GGPP normalized simvastatin-treated altered genes. Therefore, KRAS protein trafficking is successfully inhibited by statin treatment in pancreatic cancer [52].

### 3.8. Avicin G

The triterpenoid saponin avicin G (Table 1), derived from the *Acacia victoriae* plant, mislocalizes KRAS^G12V^ from the plasma membrane (PM) and disrupts the spatial organization of oncogenic K-Ras and H-Ras at the PM by lowering the contents of phosphatidylserine (PtdSer) and cholesterol at the inner PM leaflet, respectively [53]. In addition, avicin G prevents KRAS^G12V^ and HRAS^G12V^ mutations by suppressing the pERK signaling pathway and pAkt, but higher inhibition was observed in KRAS^G12V^ cells. Furthermore, human non-small cell lung cancer (NSCLC) and pancreatic ductal adenocarcinoma (PDAC) cells were used in a cell proliferation assay. Both types of cancer cell lines showed significant growth suppression. Further investigation suggests that lysosomal activity is essential for KRAS-driven cancer growth, but avicin G inhibits this by enhancing lysosomal PH and preventing phosphorylation of ERK signaling [54,55].

### 3.9. Prostratin

The phorbol ester prostratin (Table 1) was first isolated from the bark of the mamala tree of Samoa, *Homalanthus nutans* (Euphorbiaceae) [36]. Prostratin suppresses non-canonical Wnt/Ca2+ signaling, which in turn reduces the growth of KRAS and HRAS tumors. Although they have equivalent levels of canonical RAS signaling, KRAS causes more cancer than HRAS. This is due to KRAS’s ability to block Fzd8-mediated non-canonical Wnt/Ca2+ signaling, which is directly correlated with the potential to initiate tumors. The threonine 286 was phosphorylated because of the downregulation of Fzd8 and the lower CaMKii level in the KRAS^G12V^ mutation compared to HRAS^G12V^. Hence, by interrupting KRAS calmodulin-binding, prostatin (a PKC activator) elevates the level of Fzd8, which prevents *KRAS* mutation in pancreatic cancer cells [36].

### 3.10. Lupeol

Lupeol (Table 1) is a triterpenoid that inhibits FPTase and particularly inhibits KRAS mutants rather than wild-type *KRAS*. Ganaie et al. discovered 20 terpenoids after studying their crystal structures for their KRAS binding affinity. A comprehensive investigation of lup-20 (29)- en-3*b*-ol (lupeol) as a KRAS inhibitor was performed, and lupeol was identified as a powerful KRAS inhibitor using differential scanning fluorimetry, immunoprecipitation tests, and isothermal titration calorimetry. Lupeol shows its potential inhibitory effect on the KRAS^G12V^ mutant. In vivo lupeol administration inhibited the development of pancreatic intraepithelial neoplasia in mouse models [56].

### 3.11. MRTX1133

MRTX1133 is a potent, noncovalent, and selective KRAS*^G12D^* inhibitor that was reported by Wang and colleague [57]. to have anticancer activity in murine animal models through its effectiveness in deactivating KRAS*^G12D^* signaling in vivo. MRTX1133 works by binding at the switch II pocket, thereby inhibiting protein-protein interactions. A regression in tumor was also observed in Panc 04.03 xenograft models [57]. Furthermore, a study confirmed the antitumor effect of MRTX1133 to be the most prominent in autochthonous KRAS^G12D^ mouse models. Its mechanism includes its ability to inhibit ERK1/2 phosphorylation, changes in the tumor microenvironment (TME), and an increase in tumor associated macrophages [58].

## 4. RTK Inhibitor in Pancreatic Cancer

Receptor tyrosine kinases (RTKs) are transmembrane proteins expressed on the cell membrane that act as signal transducers. They are responsible for different vital cellular regulations that cause proliferation, apoptosis, differentiation, and metabolism [59]. RTK alteration happens in a wide range of cancers, emphasizing its significant role in cancer progression and as an appropriate therapeutic target. RTK comprises 58 members from 20 subfamilies. Among them, Vascular Endothelial Growth Factor Receptor (VEGF) [60] and Insulin Receptor (IGF-IR) [61] are overexpressed in pancreatic cancer.

### 4.1. Pazopanib

Pazopanib (Table 2) is a small molecule VEGF receptor (1, 2, and 3 inhibitor) and is known as an orally bioavailable drug. Phan et al. [62] studied the pazopanib effect on 52 patients, counting 32 individuals with pancreatic NETs. Among them, 21 (66%), 8 (25%), and 21 (66%) had progressive disease at enrolment, received everolimus therapy, and received previous chemotherapy, respectively. Finally, 21.9% of patients gave overall objective responses.

### 4.2. Vandetanib

The most frequently altered gene pairs in PDAC are *KRAS* and *TP53*. Kaushik et al. [78] performed thorough research to obtain novel drugs for *KRAS*- and TP53-mutated PDAC patients. They observed that vandetanib (Table 2) greatly increases the sensitivity of TP53-mutated PDAC cell lines, which highlights the therapeutic potential of vandetanib in PDAC and supports clinical decision-making for TP53-mutated PDAC patients. Vandetanib also demonstrated a direct connection between the outcomes of molecular mechanics and the prognosis of particular proteins in pancreatic tumors when multiomics, molecular dynamics, and system biology analyses of *KRAS* and TP53 mutations in PDAC were conducted. Furthermore, Middleton et al. [63] investigated the combination therapy of vandetanib and gemcitabine in 381 individuals with advanced pancreatic cancer. Vandetanib is an RTK inhibitor of VEGFR2, RET, and EGFR, all of which are responsible for cancer prognosis. Although the combination of gemcitabine and vandetanib was generally well tolerated, the results showed that adding vandetanib to gemcitabine therapy did not improve overall survival in advanced pancreatic cancer when compared to gemcitabine alone. The post-hoc analysis showed that the development of rash increases the progression-free survival of patients on vandetanib, and this supports the idea that the development of rash might be indicative of regression of pancreatic cancer.

### 4.3. Lapatinib

Lapatinib (Table 2) is a small molecule inhibitor that inhibits HER2 as well as the epidermal growth factor receptor (EGFR). Komoto et al. [79] examined the efficacy of using lapatinib either by itself or in combination with the fluoropyrimidine derivative S-1 to treat pancreatic cancer. They administered lapatinib as a single agent in vivo for four types of pancreatic cancer cell lines (MiaPaca-2, PANC-1, Capan-1, and Capan-2) to identify HER2/EGFR expressions in each of the four pancreatic cancer cells. The result showed significant suppression of tumor growth for all examined pancreatic cancer cell lines. The synergy between lapatinib and S-1 indicated inhibition of the tumor growth of MiaPaca-2 and PANC-1 xenografts. Combination therapy of lapatinib, an EGFR and HER-2 inhibitor, and capecitabine, a third-generation aromatase inhibitor (AI), showed a tolerable regimen for patients with gemcitabine-refractory pancreatic cancer [64]. However, this single-arm phase II trial was conducted for a very small number of patients. This study also reported severe side effects for 17% of patients, although there were no cardiac side effects for all the patients, and most patients tolerated their medications without dose adjustment [64].

### 4.4. Erlotinib

Erlotinib (Table 2) has shown its effect on non-small cell lung cancer (NSCLC) as a single agent. It has also been discovered that this effect is limited to patients who possess the EGFR mutation. Data from NSCLC patients demonstrated that *KRAS* mutation is very low in NSCLC but high in pancreatic cancer, and that it has a significantly lower benefit from EGFR tyrosine kinase inhibitor. El-Rayes et al. [80] identified the expression of EGFR, Akt, and NF-KB in six human pancreatic cancer cell lines. In BxPC-3, CAPAN-2, and AsPC-1 cells, combining genistein with erlotinib significantly increased (*p* < 0.05) erlotinib-induced growth inhibition and apoptosis. Significant downregulation of EGFR, phosphorylated Akt, NF-KB activation, and survivin was seen in the BxPC-3 cell line as compared to erlotinib-treated cells. Renouf et al. [65] studied 29 patients, of whom 27 (93%) were KRAS mutated, and there was no difference in outcomes when compared with the wild-type KRAS mutation. In addition, erlotinib and gemcitabine combinations were analyzed in 117 patients, and the results indicated non-significant changes. Finally, dose escalation of erlotinib might be the indicator, but it is not very effective in non-selected patients with advanced pancreatic cancer resistant to gemcitabine.

### 4.5. Axitinib

Axitinib (Table 2) is a selective and potent VEGF inhibitor and showed effectiveness in pancreatic cancer in vitro and in vivo when treated with irinotecan. By downregulating ERK1/2 and Akt phosphorylation, the combination therapy of axitinib and irinotecan showed antiproliferative and proapoptotic activity and significantly inhibited the expression of the *ATP7A* and *ABCG2* genes in endothelial and cancer cells [81]. Ioka et al. [66] investigated the efficacy of axitinib and gemcitabine combinedly in patients with advanced pancreatic cancer from different countries (Japan, North America, and the European Union). All the data suggested that, at a tolerable dose, axitinib/gemcitabine did not provide survival benefits over gemcitabine alone in patients with advanced pancreatic cancer from Japan, North America, and the European Union.

### 4.6. PD173074

PD173074 (Table 2) is a VEGF-RII and FGF-RI inhibitor targeting neoangiogenesis and mitogenesis. Treatment with PD173074 in PDAC containing the cancer cell line COLO357PL inhibited tumor angiogenesis, signal transduction, and mitogenesis. In case of in vivo study, the activity of PD173074 was reflected by a significant decrease in the intratumoral level of phospho-FGFR1 when compared to the vehicle-treated or TAE684 (ALK inhibitor)-treated mice [82]. Büchler et al. [67] were the first to identify high doses of PD173074 as a single compound and provided preclinical evidence that the small molecule inhibitor PD173074 tailored cancer therapy toward simultaneous inhibition of angiogenesis, induction of apoptosis, and inhibition of tumor growth factor-mediated mitogenesis. In an in vivo study on HPAF-II and MIA PaCa-2 cells, PD173074 showed prominent inhibition of cell growth and expressed high levels of FGF-RI.

### 4.7. Bemcentinib

Bemcentinib (Table 2) is a small molecule inhibitor that targets RTK in pancreatic ductal adenocarcinoma (PDAC). Axl signaling upregulates the TBK1–NFκβ pathway and stimulates innate immune suppression in the tumor microenvironment. In vitro analysis of six human and three mouse PDAC cell lines showed dose-dependent inhibition with IC_50_ values ranging from 1–4 µmol/L. In addition, combination treatment with Bemcentinib and gemcitabine decreases cell apoptosis, decreases cell proliferation, and reduces microvessel density in genetic KIC and syngeneic Pan02 tumors [68].

### 4.8. Imatinib Mesylate

Imatinib mesylate (IM) (Table 2) is a c-Kit-dependent tyrosine kinase inhibitor. It is reported that c-kit is overexpressed in pancreatic cancer, but it shows effectiveness in a dose-dependent manner. After investigating the mechanism of resistance of pancreatic cancer to imatinib treatment, Takayama et al. [83] reported that treatment with 5 μM imatinib failed to suppress pancreatic cancer cell growth, but it is speculated that the MEK–MAPK pathway is responsible for the resistance of pancreatic cancer to imatinib. It is also identified that combination treatment with 5 μM imatinib and 1 μM U0126 (a MEK inhibitor) significantly decreases pancreatic cancer cell growth. Imatinib mesylate is also a platelet-derived growth factor (PDGFR) that is rich in the pancreatic tumor microenvironment. Imatinib increases drug access by inhibiting interstitial fluid pressure. Moss et al. [70] investigated the effects of Imatinib mesylate and gemcitabine in phase 2 trial of forty-four patients with metastatic pancreatic cancer. Results showed that the combination of IM and gemcitabine was better tolerated than gemcitabine alone, but the overall survival rate was not statistically significant.

### 4.9. Sunitinib

Sunitinib (Table 2) is a tyrosine kinase inhibitor that targets VEGFR and PDGFR isoforms and is well known for its anti-angiogenic effect. Using an *Ela-myc* transgenic mouse model, Martínez-Bosch et al. [71] investigated the effects of sunitinib in pancreatic cancer. PDGFR and VEGFR were overexpressed in *Ela-myc* pancreatic tumors. However, treatment with sunitinib in *Ela-myc* had no impact on either early or advanced tumor progression and no effect on survival or tumor burden. However, in an in vivo study, all the subcutaneous tumors generated from tumor cell lines regressed after sunitinib treatment. All results indicated that the main barrier to sunitinib treatment in vivo is the pancreatic tumor microenvironment, which might be improved by treating in combination with drugs that disrupt tumor fibrosis. It is also reported that sunitinib decreases Akt and ERK signaling pathways following ionizing radiation in pancreatic cancer. In the in vivo study, SU11248 and radiation delayed tumor growth by 6 and 10 days, respectively, whereas combined treatment delayed tumor growth by 30 days [84].

### 4.10. Sorafenib

Sorafenib (Table 2) is a RTK inhibitor that induces apoptosis by targeting STAT3 signaling and inhibits angiogenic and RAS-dependent signaling. STAT3 is frequently activated in pancreatic cancers. It is reported that Sorafenib decreased constitutive STAT3 phosphorylation (Tyr705) as well as Mcl-1 and Bcl-xL expression in a dose-dependent manner to enhance TRAIL-mediated apoptosis in pancreatic cancer cell lines. Furthermore, sorafenib therapy improved TRAIL-induced Annexin V staining and mitochondrial cytochrome c and AIF release. Due to the Bim knockdown, it was shown to reduce caspase-3, caspase-9 cleavage, and Bax/Bak activation by sorafenib plus TRAIL [85]. A phase I trial showed that the combination therapy of sorafenib and gemcitabine had activity and was well tolerated in advanced pancreatic cancer. However, the combination therapy of sorafenib and gemcitabine did not show overall survival (OS), progression-free survival (PFS), or clinical benefit in a double-blind, phase III, randomized trial for advanced-stage pancreatic cancer patients [74].

### 4.11. Losartan

Losartan (Table 2) is an angiotensin II type 1 receptor antagonist that inhibits TGFβ1 expression and activation. However, in vivo treatment with losartan delayed, but was unable to prevent invasive and metastatic progression, as at the time of sacrifice every animal exhibited an extensive and aggressive tumor burden [86]. Murphy et al. [75] investigated the neoadjuvant therapy of FOLFIRINOX (fluorouracil, leucovorin, oxaliplatin, and irinotecan) and losartan followed by chemoradiotherapy in locally advanced pancreatic cancer. In a phase II clinical trial of losartan, an angiotensin receptor antagonist was combined with the FOLFIRINOX regimen to evaluate the margin-negative (R0) resection rate. The results suggested that the treatment with FOLFIRINOX and losartan, significantly decrease plasma TSP1 and TGF-β levels indicating a high rate of R0 resection and prolonged survival rates in LAPC [75].

### 4.12. Galunisertib

Galunisertib (Table 2) is an (ALK5) serine/threonine kinase inhibitor. It is the first oral small molecule type I transforming growth factor-beta receptor to enter clinical development. Melisi et al. [76] investigated the first-line treatment of galunisertib with gemcitabine for patients associated with unresectable pancreatic cancer. The overall result suggested that the combination therapy of galunisertib and gemcitabine increases overall survival (OS) more than gemcitabine treatment alone in unresectable pancreatic cancer. Minimal toxicity was also found with this treatment. Further evidence from biomarker analyses suggests that patients subgroups with higher levels of cytokines recruiting macrophages or regulatory T cells may benefit to a greater extent from treatment with galunisertib plus gemcitabine.

### 4.13. BMS-754807

BMS-754807 (Table 2) is a potent and reversible insulin-like growth factor 1 receptor inhibitor. Awasthi et al. investigated the therapeutic potential of combination therapy with BMS-754807 and gemcitabine in PDAC [77]. BMS-754807 and gemcitabine inhibited cell proliferation in PDAC cells, as determined by the WST-1 assay. BMS-754807 inhibited PDAC cell proliferation at 10 mmol/L of 54%, 37%, 49%, and 39% in AsPC-1, BxPC-3, MIA PaCa-2, and Panc-1 cells, respectively, in a dose-dependent manner. Additionally, it is determined that the combination therapy of BMS-754807 and gemcitabine increases the inhibitory effect more than gemcitabine alone. The addition of BMS-754807 decreased the gemcitabine IC_50_ from 9.7 mmol/L to 75 nmol/L for AsPC-1, from 3 mmol/L to 70 nmol/L for Panc-1, from 72 to 16 nmol/L for MIA PaCa-2, and from 28 to 16 nmol/L for BxPC-3 cells. BMS-754807 in combination with gemcitabine also showed strong antitumor activity by decreasing phospho-IGF-1R and phospho-AKT in tumor tissue lysates [87].

## 5. Mixed (Combined) Inhibitors of Pancreatic Cancer

Some mixed inhibitors of pancreatic cancer have been discussed, and their structures are shown in Table 3 below [88,89,90,91,92,93,94,95,96,97,98,99,100,101,102,103,104,105,106,107,108,109,110].

### 5.1. Thymidylate Synthase

#### 5.1.1. Adjuvant 5-Fluorouracil and Folinic Acid

Thymidylate synthase (TS) is an important enzyme that plays an essential role in the biosynthesis of thymidylate and replication of DNA, where it catalyzes the conversion of deoxyuridine monophosphate (dUMP) into thymidylate (dTMP), with 5,10-methylene tetrahydrofolate (CH_2_THF) acting as a methyl donor [111]. TS is a major target for cytotoxic agents, and these agents exert their inhibitory effect on TS signaling by binding either to the dUMP binding site or the CH2THF site. 5-fluorouracil (5-FU) (Table 4), a chemotherapeutic that is widely effective in the treatment of cancer, both alone and when combined with other drugs, has TS inhibition activity. It was first synthesized by Heidelberger et al. [112] in 1957 by substituting a hydrogen atom of a pyrimidine with fluorine, leading to the formation of a competitive antagonist of uracil. Inside the cell, 5-FU is converted into three main active metabolites, namely: 5- fluorouridine triphosphate (FUTP), 5-fluorodeoxyuridine triphosphate (FdUTP), and 5-fluorodeoxyuridine monophosphate (FdUMP), which are involved in RNA, DNA biosynthesis, and TS inhibition, respectively. FdUMP inhibits thymidylate synthesis by forming an inactive and stable ternary complex with TS and CH_2_THF and impeding the binding of the normal substrate, dUMP. After several clinical trials, lots of research is being conducted to determine ways in which the drug efficacy of 5-FU can be enhanced through the co-administration of agents with synergistic effects [113]. Some experimental studies have reported that an increased cellular level of CH2FH4 increased the stability of this ternary complex, thereby leading to optimal inhibition of TS. Folinic acid (FA), a precursor of CH2THF, has been shown to increase the cytotoxicity efficacy of 5FU when administered together in some clinical trials. In a study, Neoptolemos et al. [88] investigated ESPAC-1, ESPAC-1 plus, and early ESPAC-3(v1) to identify the Adjuvant 5-fluorouracil + folinic acid effect in pancreatic cancer. The result from individual patient data meta-analysis of the ESPAC-1, ESPAC-1 plus, and ESPAC-3 trials confirmed a significantly better overall survival (OS) for patients treated with 5FU/FA (HR of 0.70), which means a significant decrease in the death rate of 30% with 5FU/FA when compared to only surgery. Table 4 shows the chemical structures and corresponding biomolecular targets of other inhibitors and combine inhibitors of pancreatic cancer [114,115,116,117,118,119,120,121,122,123,124]. 

#### 5.1.2. Gemcitabine + Capecitabine

Gemcitabine is a thymidylate synthase inhibitor widely used to treat metastatic pancreatic cancer [125]. Neoptolemos et al. [89] investigated the safety and efficacy of gemcitabine and capecitabine compared to gemcitabine alone in resected pancreatic cancer. The results showed adjuvant chemotherapy with gemcitabine and capecitabine significantly increased the overall survival rate and acceptable toxicity rate in pancreatic cancer. It is also investigated that in ESPAC-4 trial, the overall survival was estimated to be 28.8% for an estimated 5 years with gemcitabine and capecitabine treatment, compared to 16.3% for gemcitabine alone and 15.9% (12.7–19.4) with 5-fluorouracil plus folinic acid in the ESPAC-1 trial [126].

### 5.2. Hedgehog Signaling

#### 5.2.1. IPI-926 (Saridegib) + Gemcitabine

The Hedgehog pathway (HhP) is an important pathway for embryonic development, tissue patterning, and wound healing. Any abnormality in its functions can lead to cancer development [127]. The two cell-membrane proteins found in Hh pathways are Patched (Ptc) and Smoothened (Smo), and these membrane proteins are regulated by the presence or absence of a hedgehog (Hh) ligand. Cell differentiation, migration, and proliferation can result from Smo-mediated signal transduction, which activates the Hh pathway. Any drugs that can inhibit or antagonize the Hh pathway may have therapeutic potential in cancer treatment [128]. Cyclopamine (11-deoxojervine) was the first compound found to inhibit Hh pathway signaling. Cyclopamine is a natural steroidal alkaloid that is isolated from the corn lily (*Veratrum californicum*). Cyclopamine can inhibit the Hh pathway due to its high affinity for Smo. However, its poor solubility and low potency prevent its clinical usage [127]. The search to improve the pharmaceutical potency gave birth to the discovery of IPI-926 (Table 3), a novel semi-synthetic analogue that inhibits the Hh pathway by binding to Smo. IPI-926 has shown an excellent oral bioavailability. It has a long plasma half-life and duration of action, and is highly distributed in tumor tissues. However, growth and maintenance of the stromal tissue in the tumor microenvironment that supports tumor growth can occur due to the paracrine mode of Hh activation [128]. In a genetically engineered murine model of pancreatic cancer, IPI-926 treatment results in depletion of tumor-associated stroma and prolonged survival when given in combination with gemcitabine. IPI-926 is currently being investigated in a phase 1 clinical trial [128]. Furthermore, the results from a genetically engineered mouse model of pancreatic cancer showed that IPI-926, when given in combination with gemcitabine, can deplete tumor-associated stromal tissue and increase intratumoral mean vessel density, which led to prolonged survival in the mouse model [129].

#### 5.2.2. IPI-269609

IPI-269609 (Table 3) is another novel, orally bioavailable small molecule that has been found to have Hedgehog inhibitory activity. PI-269609 has shown resemblance to cyclopamine in blocking Hedgehog signaling in pancreatic cancer cells in vitro and in vivo [91]. There are several mechanisms through which IPI-269609 exerts its antitumor effects. In a study by Georg et al. [91], orthotopic E3LZ10.7 xenografts were treated with IPI-269609 for 5 days using s.c. osmotic Alzet pumps, and it was shown through immunohistochemistry that IPI-269609 reduced the overexpression of ALDH (aldehyde dehydrogenase–bright cells, a clonogenic tumor-initiating population in pancreatic cancer) in vivo. The study also reported complete abrogation of distant metastases in orthotopic E3LZ10.7 xenograft male mice, after treatment with IPI-269609 (20 mg/kg/d). Furthermore, Gli-responsive (glioma-associated oncogene) reporter knockdown, down-regulation of the Hedgehog target genes *Gli1* and *Ptch* (protein patched), together with abrogation of cell migration and colony formation in soft agar, were seen in the in vitro treatment of pancreatic cancer cell lines with IPI-269609.

#### 5.2.3. Vismodegib + Gemcitabine (GDC-0449)

Gemcitabine (2′,2′-difluorodeoxycytidine) is a molecule that can induce apoptosis but has a small overall clinical potential due to its rapid metabolism by cytidine deaminase into its inactive metabolite, 2′,2′-difluorodeoxyuridine (dFdU), and excretion through the kidney [130]. A higher dose is needed to reach the therapeutic level, and this may lead to toxicity and other side effects. Thus, combination therapy is a promising approach for the treatment of pancreatic cancer. Vismodegib (GDC-0449) (Table 4) is a synthetic SMO (smoothened homolog) small molecule inhibitor of the hedgehog Hh pathway [92]. In 2016, Melek et al. [130] reported the synergistic effect between gemcitabine and GDC-0449 by preparing a micelle mixture for coadministration of both, and this showed a significant inhibition of MIA PaCa-2 xenograft tumor growth, induced apoptosis, and suppressed epithelial-to-mesenchymal transition (EMT). The Hh pathway controls epithelial and mesenchymal interactions in many tissues. The effect of some cytotoxic agents can be increased through the reversal of EMT by inhibiting Hh signaling. Sonic hedgehog signaling pathway (SHH)-driven cancers arise from a variety of mutations that affect different components, like the key transcriptional effector Gli proteins, and lead to a variety of human malignancies, including pancreatic cancer. GDC-0449 is a small molecule inhibitor of Smoothened that has been reported to induce apoptosis in three human pancreatic cancer cell lines and cancer stem cells (CSCs) as well as reduce cell visibility within 24 h after its administration, with a maximum effect seen at 72 h [131]. Furthermore, GDC-0449 inhibited Gli-DNA interaction, Gli transcriptional activity, and decreased Gli nuclear translocation in pancreatic CSCs. The downregulation of Bc1-2, the upregulation of Fas, DR4 and DR5 expressions, and a decreased PDGFR are said to be the mechanisms through which GDC-0449 induces cancerous cell death in pancreatic CSCs.

### 5.3. Mitogen-Activated Protein Kinase Kinase (MEK)

#### 5.3.1. CI-1040 (Synonym: PD184352)

The overexpression of the mitogen-activated protein kinase (MAPK) or extracellular signal-regulated protein kinase (ERK) pathway is an important therapeutic target as many key growth factors, cytokines, and proto-oncogenes transduce their signals through this pathway [132]. CI-1040 (Table 3) (cyclopropylmethyl hydroxamate ester of a *N*-phenylanthranilic acid) is the first mitogen-activated protein kinase kinase (MEK)-targeted inhibitor to enter clinical trials, and it selectively inhibits both MEK isoforms, MEK1 and MEK2. The inhibitory effect of C1-1040 on the clonogenic growth of different tumor cell lines has been shown in some preclinical studies, with the most sensitive tumors being those with high phosphorylated ERK (pERK) levels. The activation of the MAPK pathway enables the passage of cells across the G1 restriction point, and C1-1040 exhibits its antiproliferative effects by blocking G1 in a dose-dependent manner. In an in vivo study of human xenograft models (BxPc-3 and MIA PaCa-2), treated with CI-1040, a 60% rate of complete response was observed. During a preclinical evaluation phase [13], the phosphorylation of ERK by activated MEK provided a useful mechanism of MEK inhibition by C1-1040. In a period of 6 h, tumors were reduced after CI-1040 treatment, indicating a complete suppression of ERK phosphorylation; by 12 h, ERK phosphorylation started appearing, and it fully returned by 24 h after a single oral dose of CI-1040.8. Based on these findings, multiple daily dosing regimens of C1-1040 are needed to sustain suppression of MAPK phosphorylation. Furthermore, Patricia et al. [93] reported 800 mg BID as the recommended phase II dose of C1-1040 and that it should be administered with food. Out of 66 patients who were assessable for response, one partial response was shown in a patient with pancreatic cancer, and 19 patients (28%) showed stable disease lasting a median of 5.5 months. Furthermore, 10 patients demonstrated inhibition of tumor pERK (median, 73%; range, 46% to 100%).

#### 5.3.2. Pimasertib + Gemcitabine

Pimasertib (Table 3) is an orally bioavailable small molecule inhibitor of the mitogen-activated protein kinase kinase 1/2 (MEK1/2) that serves as an antitumor agent through its ability to inhibit the activation of MEK1/2- dependent effector proteins (ERK 1/2) and transcription factors [94], and it binds to an allosteric site adjacent to the ATP binding site of MEK [133]. Pimasertib has some synergistic activity when administered with gemcitabine, as it helps to increase the responsiveness of tumor cells to the effects of gemcitabine in a pancreatic cancer mouse model. In a two-part study that was conducted to investigate pimasertib in combination with gemcitabine in patients with metastatic pancreatic adenocarcinoma (mPaCa) [94], the recommended phase II dose (RP2D) of pimasertib was said to be 60 mg BID plus 100 mg/m^2^ weekly of gemcitabine. The progression-free survival (PFS) and overall survival (OS) results showed no treatment benefit for pimasertib over placebo in combination with gemcitabine (median PFS 3.7 and 2.8 months, respectively; HR 5 0.91, 95% CI: 0.58–1.42: median OS 7.3 vs. 7.6 months, respectively). Whereas, in another preclinical study [133], there was an enhanced activity of gemcitabine was observed after the models were exposed to pimasertib through significant decrease level of ribonucleotide reductase large subunit-1 (RRM1), an important gene of gemcitabine sensitivity and resistance whose expression is specifically dependent on MEK inhibition. Normally, gemcitabine binds and irreversibly inactivates ribonucleotide reductase large subunit one (RRM1) which is the rate-limiting step of DNA duplication by catalyzing the conversion of ribonucleotides into 20 -deoxyribonucleotides required for new DNA synthesis and repair. Some clinical studies [134] showed that RRM1 can act as a tumor suppressing gene while some other studies reported that its overexpression can lead to gemcitabine resistance in patients with PDAC leading to a worse overall survival [135].

### 5.4. Src Kinase

#### 5.4.1. Dasatinib + Gemcitabine

Src, one of the nine members of the Src-family kinases (non-receptor tyrosine kinases), plays a vital role in cellular proliferation, as its overexpression is associated with the progression of pancreatic neoplasia, resistance to anticancer therapies, and a poor prognosis in pancreatic cancer patients. Src plays an essential role in regulating the cytoskeleton, cell migration, adhesion, and invasion in a variety of human cancers during metastasis, and its inhibitors can be a source of promising oncologic therapeutic agents for pancreatic cancer [136]. Dasatinib (Table 3) is a potent oral Src inhibitor that has been found to inhibit pancreatic tumor growth in a xenograft mouse model but not in clinical practice. In 2019, Ling and colleagues investigated the combination efficacy of dasatinib with gemcitabine on human and mouse pancreatic cancer cells, and results showed that dasatinib can enhance the effects of gemcitabine in five pancreatic cancer cell lines and three mouse pancreatic cancer cell lines. The synergistic effect of dasatinib on gemcitabine did not only decrease the Src level but also STAT3, ERK, and p-AKT, which are tumor-associated pathways. Another phase 2 placebo-controlled trial [95], which was conducted to ascertain if the addition of dasatinib to gemcitabine would inhibit the development of metastases and improve overall survival, showed no improved overall survival in patients with locally advanced pancreatic cancer, and they accounted that this might be as a result of them using a higher dose of gemcitabine (1000 mg/m^2^) than the recommended dose of 600 mg/m^2^ and dasatinib 100 mg PO daily from the phase I study.

#### 5.4.2. Saracatinib (AZD0530) + Gemcitabine

Saracatinib (Table 3) is an orally bioavailable Src/Abl inhibitor that has shown potent antimetastasic effects in some preclinical models of tumors, including pancreatic cancer. A phase I/II study [96] of saracatinib in combination with gemcitabine in patients with advanced pancreatic cancer was conducted to determine the dosage and efficacy of saracatinib when combined with gemcitabine. Saracatinib (175 mg PO daily) was used as the RP2D in combination with gemcitabine (1000 mg/m2), but of the 22 response-evaluable patients treated, 40.9 percent had progressive disease, 27.3% had stable disease for less than 4 months, and 22.7% had SD4, while two (9.1%) had a partial response, indicating that Saracatinib (175 mg) + gemcitabine did not outperform gemcitabine alone.

### 5.5. Poly(ADP-Ribose) Polymerase Inhibitors (PARP)

#### 5.5.1. Veliparib (Synonym: ABT-888)

DNA damage repair (DDR) occurs through four different pathways, of which Base Excision Repair (BER) is one, and this BER pathway is mediated by a group of enzymes known as PARP [137]. Veliparib (Table 3) is an oral potent inhibitor of PARP1/2 that has shown single-agent preclinical and clinical activity in several germline BRCA+ cancers, including breast and prostate cancer [138]. A phase II trial [97] conducted to assess the response rate of Veliparib in patients with previously treated BRCA1/2- or PALB2-mutant (PDAC) demonstrated that Veliparib was well tolerated, but there was no significant response. The median PFS and OS were 1.7 months and 3.1 months, respectively, and 6% of the patients had an unconfirmed PR at 4 months, PD at 6 months, and 25% had stable disease.

#### 5.5.2. Olaparib (AZD2281; KU0059436)

Olaparib (AZD2281, LynparzaTM) (Table 3) is an oral inhibitor of polyadenosine 5′-diphosphoribose [poly-(ADP-ribose)] polymerization (PARP), an essential enzyme in DNA repair, being developed by AstraZeneca for the treatment of solid tumors [98]. The accumulation of single-strand breaks, which are converted during replication to irreparable DNA double-strand breaks leading to apoptosis, is due to PARP inhibition and is mostly seen in tumors exhibiting defects in homologous DNA repair. Cells with these defects, such as BRCA cells, are overly sensitive to PARP inhibition, in a process known as synthetic lethality [139]. The result of a placebo-controlled POLO (Pancreas Cancer Olaparib Ongoing) phase 3 trial, conducted to examine the effect of treating patients who had a germline *BRCA1* or *BRCA2* mutation and metastatic pancreatic cancer with Olaparib (300 mg twice daily) showed that Olaparib had a median progression-free survival of 7.4 months, compared to 3.8 months in the placebo group [140]. And after 6 months, the number of patients in the Olaparib group who became free from disease progression was more than double the number in the placebo group.

### 5.6. Transcription-3 (STAT3)

#### 5.6.1. PG-S3-001

PG-S3-001 (Table 3), a small molecule derivative of the SH-4-54 class of STAT3 inhibitors, was found to have an antiproliferative effect on pancreatic cancer cells both in vivo and in vitro [99]. STAT3 is a transcription factor that regulates stromal cell proliferation, viability, angiogenesis, metastases, and gemcitabine therapy resistance in pancreatic cancer, and several studies have shown that its inactivation can inhibit the survival and proliferation of human PDAC cells, making STAT3 a target of chemotherapeutic agents. STAT3-mediated tumorigenesis is associated with its ability to increase the levels of genes encoding antiapoptotic and proliferation-associated proteins (examples: Bcl-2, Bcl-xL, Mcl-1, Cyclin D1, and c-Myc), tumor angiogenesis inducers (VEGF and FGF), and tumor cell migration and invasion promoters (MMP-1, -2, -9, and -10) [141]. The tyrosine residue 705 (Tyr705) in the STAT3 Scr Homology2 domain is phosphorylated during its activation, causing the dimerization of STAT3 monomers, which enter the nucleus and bind at a specific DNA site, where it induces transcription of the target gene [141]. In a study [99] performed with 3D cultures of patient-derived pancreatic cancer cells, the administration of PG-S3-001 showed significant tumor growth inhibition and cell killing through its ability to potently bind (Kd = 324 nmol/L) to the STAT3 protein, thus decreasing its phosphorylation in pancreatic cancer cells.

#### 5.6.2. XZH-5

Another STAT3 inhibitor of great importance is XZH-5 (Table 3), which has been demonstrated to inhibit STAT3 phosphorylation (Tyr705), thereby leading to a decreased STAT3 activities, which resulted in inhibited colony formation, cell migration, and apoptosis induction in human pancreatic cancer cells. XZH-5 was also shown to exhibit a synergistic effect on Gemcitabine chemotherapy [100]. Lui et al. [100] reported that XZH-5 inhibited IL-6 (a mediator of inflammation and STAT3 activator) and stimulated STAT3 phosphorylation and nuclear accumulation in pancreatic and breast cancer cells. Its inhibitory effect was believed to be in its ability to suppress STAT3 downstream genes, such as Bcl-2, Bcl-xL, Cyclin D1, and Survivin.

#### 5.6.3. Cryptotanshinone

Cryptotanshinone (CPT) (Table 3), one of the active constituents in Salvia miltiorrhiza, is a fat-soluble diterpenoid anthraquinone compound that has been reported to have an antiproliferative and apoptosis-inducing effect in human pancreatic cancer cells [142]. The antitumor effect of CPT is in its inhibitory effect on STAT3, as confirmed by Yuqing et al. [102], who investigated the effect of CPT on BxPC-3 human pancreatic cells. The result confirmed suppression of STAT3 phosphorylation by CPT in a dose- and time-dependent manner. CPT only inhibited the phosphorylation of STAT3 within 30 min of exposure; however, after 24 h, CPT significantly increased the protein levels of cleaved caspase-3, caspase-9, and poly ADP ribose polymerase, while the levels of c-myc, survivin, and cyclin D1 were downregulated.

### 5.7. Hydroxamate-Based Histone Deacetylase (HDAC)

#### 5.7.1. ST-3595

ST-3595 (Table 3), an inhibitor of hydroxamate-based histone deacetylase (HDAC), exhibited anti-proliferative and cytotoxic effects on PANC-1, AsPC-1, and Mia-PaCa-2 pancreatic cancer lines and human-derived pancreatic cancer cells [103]. HDACs are a class of enzymes that downregulate the expression of tumor suppression and differentiation genes through the removal of the acetyl group of histones, thus stabilizing the DNA-histone complexes, which induce chromatin compaction [143]. HDACs are usually overexpressed in several solid tumors, like pancreatic cancer, making them an essential target of anti-cancer therapy [144]. Histone acetylation is an epigenetic process that is associated with cancer initiation and progression; increased acetylation increases transcriptional activity, while deacetylation, or decrease in acetylation, leads to silencing of gene expression. The result of a study conducted to investigate the growth inhibition and apoptosis-inducing effect of ST-3595 on pancreatic cancer cells, either alone or in co-administration with gemcitabine, showed that ST-3595 activated caspase-dependent apoptosis, inhibited proliferation, and sensitized the cytotoxic effect of gemcitabine in pancreatic cancer cells. The result suggests that the apoptosis-inducing effect of ST-3595 was due to the mitochondrial permeability transition pore (MPTP) opening [103].

#### 5.7.2. Ivaltinostat (CG200745)

The anticancer effects of ivaltinostat (Table 3), an intravenous hydroxamate-based pan-HDAC inhibitor previously referred to as CG200745, have been reported in various solid tumors like prostate cancer, cholangiocarcinoma, and pancreatic cancer [143], and it is said to induce cell death by modulating acetylation of p53, a tumor suppressor. In a complete report on a phase II clinical trial of HDACi-based chemotherapy for PDAC [143], Ivaltinostat with the gemcitabine/erlotinib regimen presented the results of Objective Response Rate (ORR 25%) and Disease Control rate (DCR 93.8%) (PR 25%, SD 68.8%) among 16 patients with an estimated OS and Progression-Free Survival (PFS) of 0.4 and 5.7 months, respectively. The ORR of 25% was much higher than the ORR of 8.6% seen in the former phase III study with a gemcitabine and erlotinib regimen. Furthermore, DCR, OS, and PFS were much better when compared to the former study. In a result from another study by Hee Seung et al. [104], a synergistic effect was seen with the co-administration of CG200745 and conventional chemotherapeutic regimens, as pancreatic cancer-cell proliferation was inhibited both in vitro and in vivo. Furthermore, the anticancer effect of CG200745 was shown to be due to its ability to increase the expression of pro-apoptotic proteins; BAX and p21 increased acetylated histone H3 level and showed IC_50_ in BxPC3 (2.4 μM), Cfpac-1 (10.7 μM), and HPAC (7.4 μM) pancreatic cell lines.

#### 5.7.3. MGCD0103 (Mocetinostat)

Treatments with MGCD0103 (Table 3), a class I-selective HDACI, resulted in dose-dependent growth arrest, cell death/apoptosis, and cell cycle arrest in the G2/M phase, accompanied by induction of p21 and DNA double-strand breaks (DSBs) [145]. In contrast, MC1568 (a class IIa-selective HDACI) and Tubastatin A (a HDAC6-selective inhibitor) showed minimal effects. When combined simultaneously, MC1568 significantly enhanced MGCD0103-induced growth arrest, cell death/apoptosis, and G2/M cell cycle arrest, while Tubastatin A only synergistically enhanced MGCD0103-induced growth arrest. Although MC1568 or Tubastatin A alone had no obvious effects on DNA DSBs and p21 expression, their combination with MGCD0103 resulted in the co-operative induction of p21 in the cells.

### 5.8. Bcl-2/Bcl-xL

#### 5.8.1. UMI-77

B-cell lymphoma 2 (Bcl-2) family are proteins that regulate the mitochondrial outer membrane permeabilization (MOMP) and there are two types: anti-apoptotic (Bcl-2, Bcl-X_L_, Mcl-1, A1, Bcl-w, and Bcl-B) and pro-apoptotic proteins (Bid, Bik, Bim, Bad, Puma, and Noxa). A balance between the different Bcl-2 proteins determines if mitochondria remain intact or become permeabilized and release proteins that promote apoptosis. The anti-apoptotic proteins, which are the main negative regulators of the apoptotic process as they prevent cell death, have been found to be overexpressed in a variety of aggressive human cancers, including pancreatic cancer, and their blockade is a novel therapeutic target for pancreatic anticancer agents [108]. UMI-77 (Table 3), a naphthol derivative small molecule inhibitor of Mcl-1 that selectively binds to its BH3-binding groove with *K*_i_ 0.49 μM, has been reported to induce apoptosis in a time- and dose-dependent manner in pancreatic cancer cells and inhibit tumor growth in a BxPC-3 xenograft model [107,146]. Its ability to cause cytochrome c release and caspase-3 activation, heterodimerization of Mcl-1/Bax, and Mcl-1/Bak blockade, thus antagonizing Mcl-1 function, has been confirmed as the mechanism behind its apoptotic induction [146].

#### 5.8.2. TW-37

TW-37 (Table 3), a benzenesulfonyl derivative that was first synthesized at the University of Michigan, has been found to have both pro-apoptotic and antiangiogenic effects [147]. It has high affinities for antiapoptotic proteins (Bcl-2, Bcl-X_L_, and McI-1) and targets their elongated grooves that bind to the BH3 domain of proapoptotic effectors. Asfar et al. (2008) studied [147] the effect of TW-37 with gemcitabine on growth and apoptotic processes in BxPC-3 and Colo-357 cell lines, and the result showed that TW-37 was sensitized to the cytotoxic effect of gemcitabine on pancreatic cancer apoptotic processes. Another study reported the mechanism by which TW-37 elicits its action using BxPC-3 and Colo-357 pancreatic cancer cells, which had high levels of Bcl-2, Bcl-xL, and Mcl-1. TW-37 was able to induce apoptotic cell death in both cell lines by blocking Bcl-2, which increased cell population in the S phase and CdkI proteins while decreasing levels of cyclin D1, cyclin A, and Cdk4 [108].

#### 5.8.3. ABT-737

ABT-737 (Table 3) is a small molecule BH3 mimetic that binds to and antagonizes only Bcl-2/Bcl-x_L_ antiapoptotic proteins, excluding Mcl-1. A study [104] investigated the synergistic effect of ABT-737 on tumor necrosis factor–related apoptosis-inducing ligand (TRAIL)-mediated cytotoxicity in human pancreatic cancer cell lines. ABT-737 improved TRAIL-induced apoptosis through activation of caspase-8 and Bid, cleavage of caspase-3 and PARP, enhanced Bax conformational stability, and impeded the interaction between Bak and Bcl-x_L_ in both PANC-1 and BxPC cell lines. Furthermore, ABT-737 untethered the sequestration of Bim with Bcl-2 and Bcl-x_L_.

## 6. Other Promising Inhibitors of Pancreatic Cancer

### 6.1. Topoisomerase I

#### Nab-Paclitaxel + Gemcitabine

The combination of gemcitabine and Nab-*paclitaxel* (Table 4), which is a form of paclitxal drug produced by addition of the protein molecule albumin, was approved on September 6th by the U.S. Food and Drug Administration (FDA) for metastatic pancreatic cancer [114]. Binding paclitaxel to albumin eliminates the need for solvents that keep paclitaxel soluble once injected into the body, but that can also cause allergic reactions and side effects. Binding of albumin to paclitaxel is essential, as it helps to transport paclitaxel to tumor cells and also eliminates the use of solvents that keep paclitaxel soluble in the body, as there are side effects and allergic reactions associated with their use. A phase 3 study [114] investigated the efficacy and safety of the co-administration of Nab-paclitaxel and gemcitabine versus gemcitabine monotherapy in patients with metastatic pancreatic cancer. The results showed a median overall survival of 8.5 months (nab-paclitaxel–gemcitabine group) and 6.7 months (gemcitabine group). Furthermore, the survival rate after one year and two years was 35% and 9% (nab-paclitaxel–gemcitabine group) and 22% and 4% (gemcitabine group), respectively. The median values of progression-free survival were 5.5 months (nab-paclitaxel–gemcitabine group) and 3.7 months (gemcitabine group).

### 6.2. Protein Phosphatase 2A

#### LB-100

The result of a study conducted to determine the safety, tolerability, and potential anticancer activity of LB-100 (Table 4) stated that the recommended phase II dose of LB-100 is 2.33 mg/m^2^ daily for 3 days every 3 weeks as two patients out of 29 had dose-limiting toxicity (3.1 mg/m^2^). Six patients had adverse events: anemia (*n* = 2), decreased creatinine clearance, dyspnea, hyponatremia, and lymphopenia. And out of 20 response-evaluable patients, 50% experienced stable disease for more than four cycles. And after 10 cycles, a partial response was seen in one patient with pancreatic adenocarcinoma, and this was maintained for an extra five cycles [115]. Another report [148] showed that LB-100 inhibited PP2A and sensitized human pancreatic cell line cultures and xenografts to radiation through interference with DNA repair.

### 6.3. Proteasome

#### Marizomib + Vorinostat

The result of a phase 1 clinical trial [116] conducted to ascertain the safety and synergistic effect of Marizomib (proteasome inhibitor) with Vorinostat (HDAC inhibitor) showed that 61% of evaluable patients had stable disease, while a decrease in tumor size was seen in 39%, with RP2D established as 0.7 mg/m_2_ of marizomib and 300 mg/day of vorinostat. Co-administration of both drugs did not affect the individual pharmacokinetics or pharmacodynamics. Proteasome inhibitors were suggested to induce apoptosis in cells by inhibiting the degradation of key proapoptotic and anti-growth proteins while increasing the levels of proteotoxic proteins and protein aggregation that enhance cellular stress. Another synergistic effect of the two compounds is the ability of the HDAC inhibitor to prevent aggresome (accumulated ubiquitin-conjugated proteins) formation, which is usually formed as a cytoprotective mechanism during proteasome inhibitor treatment [116].

### 6.4. mTOR

#### Everolimus

Everolimus (Evr) (Table 4) is an inhibitor of the mammalian target of rapamycin (mTOR) that modulates glucose metabolism, tube formation, cell migration, and apoptosis in pancreatic cancer cells by inhibiting the activation of the PI3K/AKT/mTOR signaling pathway. It is a known fact that Gemcitabine (GEM) resistance is a major issue in pancreatic cancer treatment; therefore, lots of research is being conducted to offer ways in which this issue can be combated. The therapeutic effect of everolimus in targeting GEM resistance in pancreatic cancer was investigated by Jing and colleagues [149]. The result demonstrated that Evr downregulated mTOR, inhibited aerobic glycolysis, and reduced the expression of glucose transporter 1, lactate dehydrogenase-B, hexokinase 2, and pyruvate kinase M2 in GEM-sensitive and GEM-resistant cells. Evr also increased Bax and cytochrome-c levels while decreasing Bcl-2 levels.

### 6.5. Thioredoxin-1 (Trx-1)

#### PX-12

PX-12 (Table 4), a novel small molecule irreversible inhibitor of the proto-oncogene Thioredoxin-1 (Trx-1) and increased levels of Trx-1 (40–50%) are associated with shorter survival in different cancers, including pancreatic, breast, and colorectal cancers. The effect of PX-12 was studied in patients with advanced pancreatic cancer who had previously been treated with gemcitabine (APC); [118] found that PX-12 was well tolerated, but treatment was ineffective in APC patients who had previously been treated with gemcitabine, and table disease was observed in only 2/16 patients. Therapy administration did not show a consistent decrease in SUV, Trx-1 levels, or CA 19-9 with a PFS of >4 months, a median PFS of 0.9 months, and survival of 3.2 months. However, the author terminated the research due to low antitumor activity and Trx-1 levels.

### 6.6. Polo-Like Kinase 1 (PLK1) Phosphoinositide 3-Kinase (PI3K)

#### Rigosertib + Gemcitabine

The synergistic effect and safety of rigosertib (ON-01910.Na) Table 4, a non-ATP-binding small molecule inhibitor of both PI3K and PLK1 pathways, was investigated when co-administered with gemcitabine in a phase II/III trial [119], and the report demonstrated no survival or response improvement was seen when compared to gemcitabine single treatment in patients with metastatic pancreatic cancer. The median OS and PRR of Rig+Gem, were 6.1 months and 19%, while those of Gem were 6.4 months and 13%, respectively. Rigosertib impedes both Plk1-mediated G_2_–M cell-cycle transition and PLK-induced gemcitabine resistance in pancreatic cancer cells [150]. And it works by inhibiting Ras activity through binding to the Ras-binding domain of effector kinases like Raf and PI3K, causing their deactivation.

### 6.7. Ataxia Telangiectasia and Rad3-Related Protein (ATR)

#### Ceralasertib (Synonym: AZD6738)

Ataxia Telangiectasia and Rad3-related (ATR), a serine/threonine protein kinase involved in the DNA damage response (DDR), cell cycle checkpoint, and serving as a sensor of replication stress (RS), is usually overexpressed in cancer due to activation of oncogenes and impairment of G1 checkpoint control [151]. Therefore, its inhibitors have potential as cancer therapeutics. AZD6738 (Table 4) was the first oral ATR inhibitor to enter clinical trials [152]. A study reported the effectiveness of AZD6738 in inducing cell death in olaparib-treated ATM-deficient cancer cells while exerting a low effect on ATM-proficient control cells. They suggested that the co-administration of PARP and ATR inhibitors may be useful for the treatment of cancer types that show deficiency in ATM [153].

### 6.8. BET Bromodomain

#### JQ1

JQ1 (Table 4) is a selective, small molecule inhibitors of BET (bromodomain and extra terminal) proteins that impedes their binding ability to acetylated lysines on histones. BET proteins modulate the activities of genes involved in inflammation, the cell cycle, and apoptosis. The anti-tumor effect of JQ1 was confirmed by Garcia et al. [154], where it was reported that the treatment of tumor-bearing mice with 0 mg/kg of JQ1 inhibited the growth of all the tumor-graft models (*p* < 0.05) containing the *KRAS* mutation. JQ1 treatment also downregulated the levels of CDC25B but did not induce changes in c-Myc protein expression. It was also seen that bromodomain inhibitors also downregulated protein levels of p-Erk 1/2 and p-STAT3 in mouse models of pancreatic cancer [155], thereby inhibiting the growth of cancer cells.

### 6.9. MDM2 Inhibitor

#### MI-319 + Cisplatin

MI-319 (Table 4) is a small molecule inhibitor of murine double minute 2 (MDM2) that restores the activities of p53 by impeding the MDM2-p53 interaction. P53 helps to induce apoptosis and regulate the cell cycle; however, its mutation is seen in pancreatic cancer cells. A study reported for the first time that, when combined with cisplatin, MI-319 induced cell growth inhibition and apoptosis in pancreatic cancer cells regardless of the mutational status of their p53, as reduced tumor growth was observed in both wt-p53 and mut-p53 tumor xenograft models. It was also observed that the inhibition of MDM2 led to the reactivation of p73 and p21, which are useful in increasing cell death [150].

### 6.10. Nuclear Factor Kappa Beta (NFκβ)

#### Spongiatrol

The activation of the Nuclear Factor Kappa Beta (NFκβ) a signal transducer, promotes metastasis in pancreatic cancer by initiating the signal cascade of pro-inflammatory cytokines. Spongiatriol (Table 4), a marine furanoditerpenoid isolated from Great Barrier Reef sponges of the genus Spongia [156] in the 1970, was reported in 2013 by Guzman and colleagues [123] to have an inhibitory effect on NFκβ transcriptional activity in a reporter cell line. The study also showed that levels of activated NFκβ in the AsPC-1 cell line were reduced, and a modest but significant apoptotic effect was induced in both the AsPC-1 and Panc-1 cell lines with spongiatriol IC_50_ NFκβ inhibition of 3.4 μM.

### 6.11. Gamma Secretase

#### MRK-003

**Figure 1:**
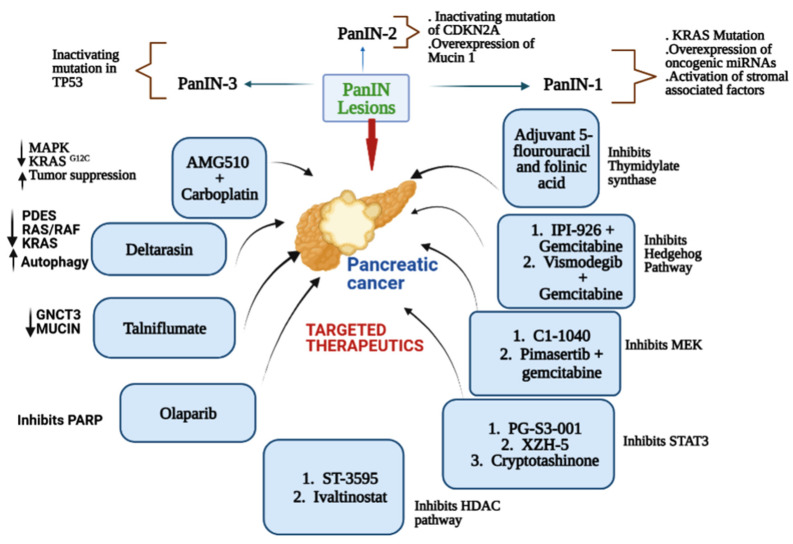
Representative diagram mentioning the major pathways of a few leading small molecule inhibitors against pancreatic cancer.

The small molecule inhibitor of γ-secretase (GSI) MRK-003 (Table 4) has been shown to efficiently block Notch activity, reducing the growth of some tumor cell lines. Notch signaling, a pathway that plays an important role in cellular development, has been linked to several human cancers, as it was found to be overexpressed in PDAC cells compared to normal cells [61]. When the Notch pathway is activated, the Notch receptor is cleaved by γ-secretase, resulting in the release of the Notch intracellular domain into the nucleus. Several studies have reported that the downregulation of Notch receptors by GSI in human pancreatic cancer cells increased apoptosis and decreased cell proliferation and invasion. In an in vivo study [124], treatment with MRK-003 led to decreased levels of the nuclear Notch1 intracellular domain, tumor-initiating cell levels, and anchorage-independent growth. PDAC xenografts treated with MRK-003 showed 56% tumor growth blockade, and co-administration of MRK-003 with gemcitabine increased antitumor effects compared with gemcitabine alone (44%). A representative diagram mentioning the major pathways of a few leading small molecule inhibitors (standalone and in combination) for pancreatic cancer is shown in Figure 1.

## 7. NRF2 Role in KRAS-Driven Pancreatic Cancer

One of the major drawbacks in the treatment of pancreatic cancer is its resistance to chemotherapy. KRAS, which has previously been identified as one of the major drivers of pancreatic cancer, upregulates the activities of NRF2-dependent chemoresistance. NRF2 is a transcription factor that regulates oxidative stress and genes involved in drug detoxification and cytoprotection. High levels of NRF2 alter glucose and glutamine metabolism by increasing glutaminolysis, which is needed for PDAC cell proliferation. Therefore, any measure taken toward decreasing glutaminolysis will aid in arresting PDAC chemoresistance [157]. It has been reported that in KRAS-PDAC, an increased level of NFR2 was observed in higher-grade lesions, and the overexpression of these NRF2s led to increased gemcitabine resistance [158]. The co-administration of CB-839, a glutaminase inhibitor, showed a synergistic effect and increased sensitivity of PDAC to gemcitabine in mouse models [159]. Another study confirmed that the use of an NRF2 activator together with a glutaminase inhibitor could offer a novel therapy for the treatment of KRAS-dependent pancreatic cancer [160]. In order to maintain anabolic cancer metabolism, KRAS mutant pancreatic cancer rewires several metabolic pathways, and an understanding of how this metabolic reprogramming works can help offer some therapeutic interventions. Aside from glucose and glutamine metabolism, KRAS also alters autophagy, mitophagy, and micropinocytosis. [161]. Through deregulating autophagy, tumor cells are protected from oxidative stress and nutrient starvation. Upregulation of micropinocytosis by PDAC cells increases proteolysis, which elevates the quantity of amino acids that are needed to support the growth of PDAC cells during glutamine restrictions [57].

## 8. Conclusions

It is well known that pancreatic cancer is a leading cause of cancer-related deaths worldwide, mainly because it is hard to detect in its early stage and several of its risk factors are out of control. Accordingly, the discovery of highly efficient drugs with higher potency and lower toxicity is timely and challenging. This review outlines the efforts that have been made in this field to date. Our discussion is focused on the small molecule inhibitors (individual and combined), both natural and synthetic, and many of these are in the investigational stage. This article critically oversees the general molecular mechanism of pancreatic cancer, with an emphasis on the discussion of the molecular mechanisms of small-molecule inhibitors (if reported). Natural and small synthetic molecules that show promise have been identified for discussion. For several molecules under discussion, only in vitro data are available, and they are far from the clinical trial stage. Based on the available literature, we have included these molecules as much as possible so that the drug development community may consider these molecules (in particular, the pharmacophores present in the small molecules) for further research. The major significance of showing the chemical structures is to help the readers identify the possible pharmacophores at a glance. Appropriate identification of the pharmacophores will help the readers in deciding on further functionalization/chemical modification, and/or semi-synthesis (for natural compounds) in developing better inhibitors by computer-assisted drug design. Any omission is purely unintentional. Additional effort has been put into comparative studies on single and combined therapies. Based on this discussion, it seems like the development of mutual or reciprocal small molecule prodrugs might be more effective in reducing the mortality rate in pancreatic cancer, although more research in this aspect is necessary before drawing a conclusion. At the same time, it is important to spread the message around the globe concerning the relationship between pancreatic cancer and lifestyle. For example, tobacco smoking is attributable to about 25% of PC cases [58]. In summary, potent small molecule drugs and a healthy lifestyle might be the best weapons in the battle against pancreatic cancer, the most fatal cancer to date.

## Figures and Tables

**Table 1:** RAS inhibitors in pancreatic cancer.

Inhibitors	Structure	Target	Ref
AMG 510 (Lumakras or Sotorasib)	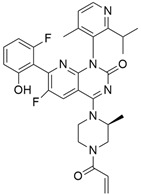	KRAS^G12C^ inhibitor	[29]
MRTX849 (Adagrasib)	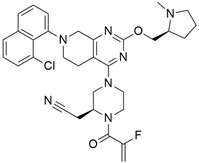	KRAS^G12C^ inhibitor	[30]
KRAS inhibitor 11 ^†^	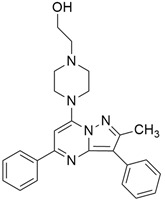	Inhibits MAPK/RAF signaling,	[31]
Deltarasin	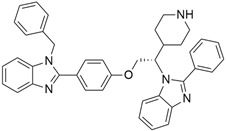	Downregulates RAS/RAF signaling pathway.	[32]
Talniflumate + Ggefitinib	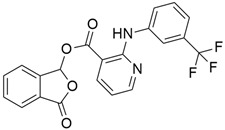	Inhibition of 2 β-1,6 N-acetylglucosaminyltransferase (GCNT3)	[33]
MDC-1016	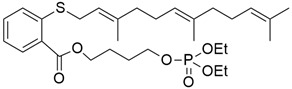	Ras inhibitor	[34]
	Natural Product Inhibitors		
Simvastatin	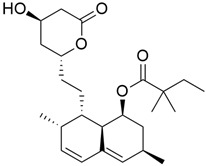	GFP-K-Ras protein trafficking	[35]
Avicin G	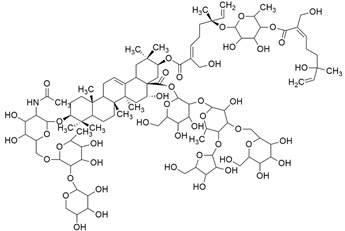	KRAS^G12V^ and HRAS^G12V^	[36]
Prostratin	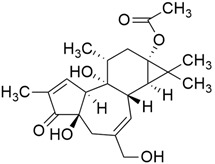	KRAS	[37]
Lupeol	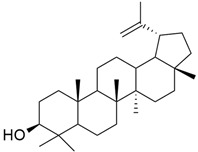	KRAS^G12V^	[38]

^†^ 2-[4-(2-Methyl-3,5-diphenylpyrazolo [1,5-a]pyrimidin-7-yl)piperazin-1-yl]ethanol.

**Table 2:** RTK inhibitors in pancreatic cancer.

Inhibitor	Structure	Target	Ref
▪ Pazopanib	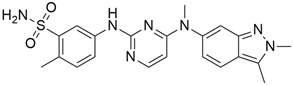	VEGF receptors 1, 2, and 3	[62]
▪ Vandetanib	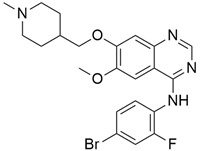	VEGFR2, RET, and EGFR	[63]
▪ Lapatinib + Capecitabine	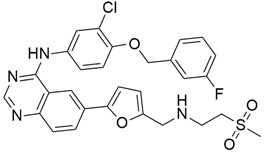	EGFR and HER-2	[64]
▪ Erlotinib + Gemcitabine	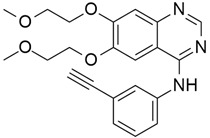	EGFR	[65]
▪ Axitinib + Gemcitabine	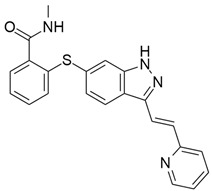	VEGFR1, VEGFR2, VEGFR3 and PDGFRβ	[66]
▪ PD173074	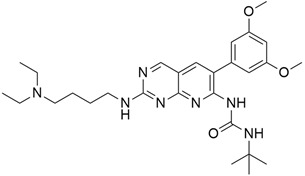	VEGF-RII and FGF-RI	[67]
▪ Bemcentinib (Synonyms: R428; BGB324)	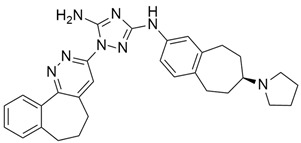	Axl	[68,69]
▪ Imatinib Mesylate	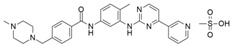	PDGFR, c-Kit, v-Abl	[70]
▪ Sunitinib	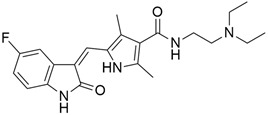	PDGFR, FLT3, IRE1α, Kit	[71]
▪ Cetuximab+ Gemcitabine	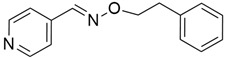	EGFR	[72]
▪ Bevacizumab+ Gemcitabine	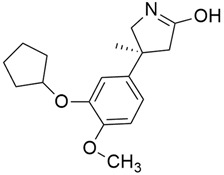	VEGFR	[73]
▪ Sorafenib+ Gemcitabine	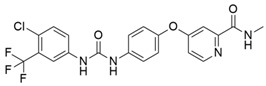	VEGFR-2/PDGFR-beta	[74]
▪ Losartan+ FOLFIRINOX	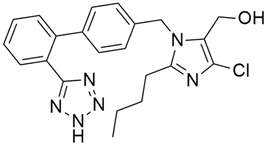	Angiotensin Receptor	[75]
▪ Galunisertib	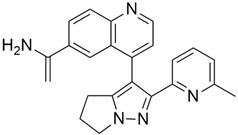	TGF-β type I receptor (TβRI)	[76]
▪ BMS-754807	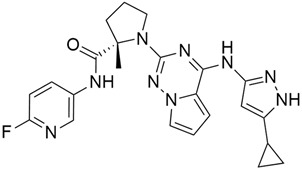	insulin-like growth factor-1R/IR	[77]

**Table 3:** Mixed inhibitors of pancreatic cancer.

	Thymidylate Synthase	
Inhibitor	Structure	Ref
Adjuvant 5-fluorouracil + folinic acid	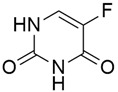	[88]
Gemcitabine + Capecitabine	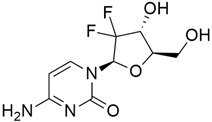	[89]
	**Hedgehog signaling**	
**Inhibitor**	**Structure**	**Ref**
IPI-926 (Saridegib) + gemcitabine	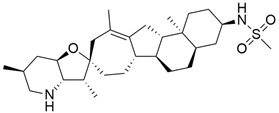	[90]
IPI-269609	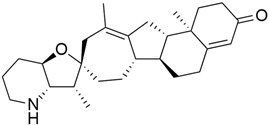	[91]
Vismodegib (GDC-0449) + Gemcitabine	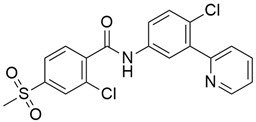	[92]
	**Mitogen-activated protein kinase kinase(MEK)**	
**Inhibitor**	**Structure**	**Ref**
CI-1040 (Synonyms: PD 184352)	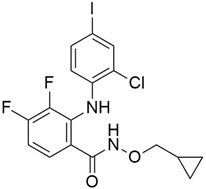	[93]
Pimasertib + gemcitabine	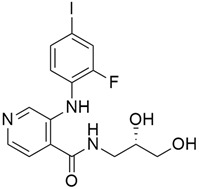	[94]
	**Src kinase**	
**Inhibitor**	**Structure**	**Ref**
Dasatinib + Gemcitabine	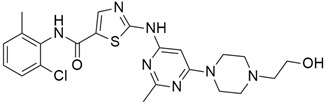	[95]
Saracatinib (AZD0530) + Gemcitabine	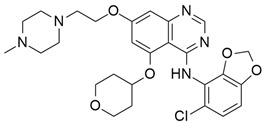	[96]
	**Poly(ADP-ribose)** **polymerase inhibitors (PARP)**	
**Inhibitor**	**Structure**	**Ref**
Veliparib (Synonyms: ABT-888)	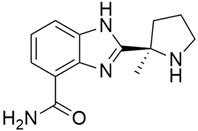	[97]
Olaparib (AZD2281; KU0059436)	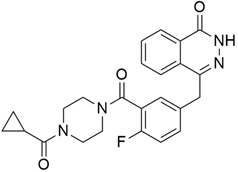	[98]
	**Transcription-3 (STAT3)**	
**Inhibitor**	**Structure**	**Ref**
PG-S3-001	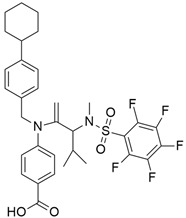	[99]
XZH-5	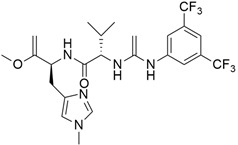	[100]
Cryptotanshinone	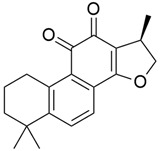	[101,102]
	**hydroxamate-based** **histone deacetylase (HDAC)**	
**Inhibitor**	**Structure**	**Ref**
ST-3595	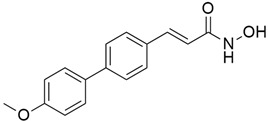	[103]
Ivaltinostat (CG200745)	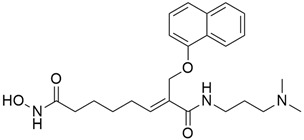	[104]
MGCD0103 (Mocetinostat)	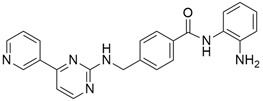	[105,106]
	**Bcl** **-2/Bcl** **-xL**	
**Inhibitor**	**Structure**	**Ref**
UMI-77	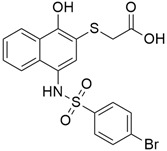	[107]
TW-37	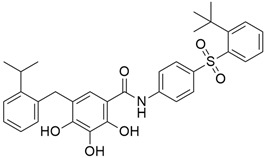	[108,109]
ABT-737	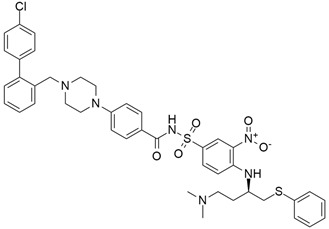	[110]

**Table 4:** Other inhibitors of pancreatic cancer.

Inhibitor	Structure	Target	Ref
nab-Paclitaxel + gemcitabine	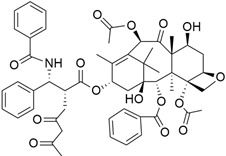	Topoisomerase I	[114]
LB-100	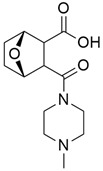	Protein Phosphatase 2A	[115]
Marizomib + Vorinostat	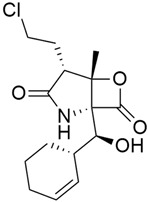	Proteasome	[116]
Everolimus	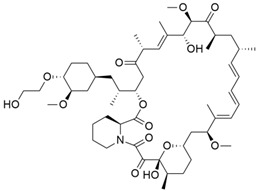	mTOR	[117]
PX-12	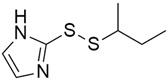	Thioredoxin-1 (Trx-1)	[118]
Rigosertib + Gemcitabine	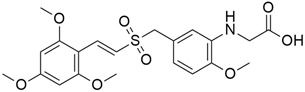	polo-like kinase 1 (PLK1) phosphoinositide 3-kinase (PI3K)	[119]
Ceralasertib (Synonyms: AZD6738)	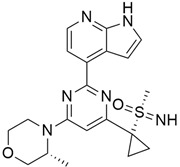	Ataxia telangiectasia and Rad3-related protein (ATR)	[120]
JQ1	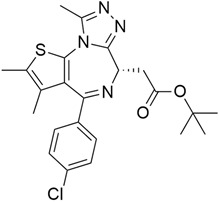	BET bromodomain	[121]
MI-319+ Cisplatin	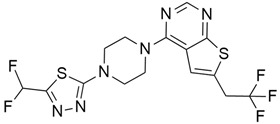	MDM2 inhibitor	[122]
Spongiatriol	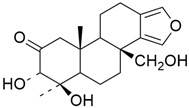	Nuclear Factor Kappa B Nuclear Factor Kappa B	[123]
MRK-003	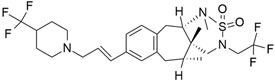	Gamma Secretase	[124]

## Data Availability

Data available in the public domain. No confidential/unpublished data have been used in this article.
